# Prevalence of Rotavirus Genotypes Among Children Under Five in Central Asia: A Systematic Review and Meta-Analysis of Available Evidence

**DOI:** 10.3390/vaccines14080668

**Published:** 2026-07-31

**Authors:** Yerke Suleimenova, Indira Karibayeva, Shnara Svetlanova, Nurzhamal Akhmadyar, Dinagul Bayesheva, Gulbanu Boranbayeva, Ainur Ikhambayeva, Saule Burkitbayeva, Aida Bolatbek

**Affiliations:** 1 Department of Clinical Pharmacology, Astana Medical University, Astana 010000, Kazakhstan; 2 Department of Health Policy and Community Health, Jiann-Ping Hsu College of Public Health, Georgia Southern University, Statesboro, GA 30460, USA; 3 Department of Nursing, Kazakhstan Medical University “KSPH”, Almaty 050004, Kazakhstan; 4 Department of Pediatric Infectious Diseases, Astana Medical University, Astana 010000, Kazakhstan; 5 Research Institute of Preventive Medicine Named After Academician E.D. Dalenov, Astana 010000, Kazakhstan; gulbanuboranbaeva2@gmail.com

**Keywords:** rotavirus, genotype distribution, children under five, Central Asia, vaccination, public health surveillance, rotavirus vaccines, strain replacement, systematic review, meta-analysis

## Abstract

**Background/Objectives:** Rotavirus remains a major cause of acute gastroenteritis among children under five years of age, particularly in regions with heterogeneous vaccine implementation. In Central Asia, differences in the timing and coverage of rotavirus vaccination may influence circulating genotype patterns, yet regional evidence remains fragmented. **Methods:** A systematic review and meta-analysis were conducted in accordance with PRISMA 2020 guidelines and registered in PROSPERO (CRD420251274198). Electronic databases (PubMed, Web of Science, ScienceDirect, and Google Scholar) were searched without date restrictions. Studies reporting laboratory-confirmed rotavirus infection and genotype distribution among children under five years in Central Asia were included. Random-effects meta-analysis of proportions was performed to estimate the pooled genotype proportions among rotavirus-positive children, with subgroup analyses comparing the before and after vaccine introduction periods. **Results:** Six studies (ten before vaccine introduction strata and four after vaccine introduction strata; N = 1713 rotavirus-positive children) were included. A significant decline in the G1P[8] genotype was observed following vaccine introduction, with pooled prevalence decreasing from 0.468 (95% CI: 0.363–0.577) to 0.068 (95% CI: 0.034–0.133) (*p* < 0.001). In contrast, G2P[4] increased from 0.134 to 0.485 (*p* < 0.001), while G9P[4] and G9P[8] also increased significantly. G3P[8] and mixed genotypes declined, whereas G4P[8], G12P[8], and untypable strains showed no statistically significant subgroup differences. Substantial heterogeneity was observed (I^2^ > 90%), and evidence certainty was very low for all genotype outcomes. **Conclusions:** The available evidence suggests that rotavirus genotype distribution among children under five in Central Asia may have changed after vaccine introduction, with lower pooled proportions of G1P[8] and higher proportions of selected heterotypic genotypes in after vaccine introduction strata. Strengthened molecular surveillance using standardized genotyping protocols is needed to determine whether these patterns represent sustained genotype replacement or temporal fluctuation. These estimates are based on a small, heterogeneous evidence base with very low certainty and should be considered hypothesis-generating rather than confirmatory of a causal vaccine effect.

## 1. Introduction

Rotavirus remains a leading cause of severe acute gastroenteritis (AGE) and diarrheal morbidity among children under five years of age worldwide, despite substantial progress in vaccine development and implementation [1,2]. Prior to the introduction of rotavirus vaccines, nearly every child globally experienced at least one episode of rotavirus infection by the age of five, with a disproportionate burden observed in low- and middle-income regions [1]. Although global mortality attributable to rotavirus has declined significantly following widespread immunization efforts, rotavirus-associated morbidity continues to exert a considerable public health burden, particularly in regions with heterogeneous vaccine uptake and limited surveillance infrastructure [2,3]. Central Asia represents a region of strategic epidemiological importance due to its transitional health systems, variable implementation of rotavirus vaccination programs, and limited consolidated evidence on circulating rotavirus strains [4,5]. Understanding regional patterns of rotavirus epidemiology is critical, as vaccine effectiveness and long-term impact are closely linked to genotype diversity and potential strain replacement dynamics [3,6]. Rotavirus genotypes are classified based on two outer capsid proteins, VP7 (G types) and VP4 (P types), which define strain-specific immunity and vaccine responsiveness. Globally, genotypes such as G1P[8], G2P[4], G3P[8], and G9P[8] have historically predominated; however, shifts in genotype distribution have been observed following vaccine introduction, raising concerns regarding vaccine-driven selective pressures and the emergence of non-vaccine strains [7,8]. Evidence from various regions suggests that post-vaccination landscapes may be characterized by declines in vaccine-covered genotypes alongside relative increases in previously less dominant strains [6,9]. Despite the public health relevance of these dynamics, the epidemiology of rotavirus infection in Central Asia remains insufficiently synthesized. Moreover, there is a lack of pooled quantitative estimates that would enable regional comparisons and assessment of genotype-specific trends before and after vaccine introduction.

### Study Context

Following Uzbekistan’s introduction of rotavirus vaccine in 2014, Tajikistan introduced the vaccine in 2015, while Kyrgyzstan and Turkmenistan introduced it in 2019 [10]. Despite data from modelling studies suggesting that the rotavirus vaccine is cost-effective and significantly reduces disease burden, Kazakhstan has not yet implemented it in its national immunisation programme [11,12].

Because Kazakhstan has not introduced the vaccine, and the Kyrgyz and Kazakh data included in this review predate 2019, the findings for these countries in this review reflect only the pre-vaccination epidemiological landscape (see Section 4.4). Overall, these differences in the timing of introduction and vaccine coverage across Central Asian countries may significantly influence rotavirus epidemiology and the distribution of circulating genotypes in the region. Therefore, the present systematic review and meta-analysis aims to synthesize available evidence on the prevalence and genotype distribution of rotavirus infection among children under five years of age in Central Asia. Specifically, this study seeks to (1) estimate pooled prevalence of rotavirus genotypes, (2) compare genotype distribution before and after vaccine introduction, and (3) evaluate the robustness and certainty of the available evidence [13,14]. The findings of this study are intended to inform regional surveillance strategies, guide vaccine policy decisions, and contribute to the broader understanding of rotavirus epidemiology in underrepresented settings.

## 2. Materials and Methods

### 2.1. Search Strategy

The review protocol was prospectively registered in the International Prospective Register of Systematic Reviews (PROSPERO; registration number CRD420251274198). Prior to conducting the review, the PROSPERO database was searched to identify any previously registered or ongoing systematic reviews addressing rotavirus prevalence and genotype distribution among children under five years of age in Central Asia. No overlapping reviews were identified. The systematic review was conducted and reported in accordance with the Preferred Reporting Items for Systematic Reviews and Meta-Analyses (PRISMA 2020) guidelines [15]. A comprehensive literature search was performed by two independent reviewers in the following electronic databases: PubMed, Web of Science, ScienceDirect and Google Scholar. Google Scholar was searched using Advanced Search with the predefined keyword combination restricted to article titles. The search returned 20 records, all of which were screened. All potentially relevant records retrieved from the search were screened based on title, abstract, and full-text assessment when applicable. The search covered all available records up to the date of the final search, with no restrictions on publication year. Only studies published in English were included. The search strategy combined Medical Subject Headings (MeSH), where applicable, and free-text terms related to rotavirus infection, prevalence, genotype distribution, and Central Asian countries. The search strategy was adapted for each database using combinations of the terms “rotavirus”, “prevalence”, “genotype”, “Central Asia”, “Kazakhstan”, “Uzbekistan”, “Kyrgyzstan”, “Tajikistan”, and “Turkmenistan”. Reference lists of included studies were also manually screened to identify additional relevant publications. Regional-language databases and grey literature sources were not systematically searched. Only peer-reviewed studies published in English were considered eligible for inclusion. The final literature search was conducted on 25 April 2026.

### 2.2. Eligibility Criteria

Studies were considered eligible if they investigated rotavirus infection among children under five years of age in one or more Central Asian countries, including Kazakhstan, Uzbekistan, Kyrgyzstan, Tajikistan, and Turkmenistan. Eligible studies were required to report laboratory-confirmed rotavirus infection and provide data on rotavirus prevalence and/or genotype distribution. Studies were eligible for the genotype meta-analysis only if they reported genotype-specific counts among laboratory-confirmed rotavirus-positive children under five. Both hospital-based and community-based studies were included to ensure comprehensive representation of the target population. Studies conducted during the pre-vaccination, transition, or post-vaccination phases were eligible, allowing for comparison across different stages of vaccine implementation. For the primary subgroup analysis, a country-period was classified as post-vaccination only when data collection began at least 12 months after the national vaccine introduction date. Data collected during the first 12 months after vaccine introduction were classified as transition-period observations. These observations were retained in the primary analysis within the broader after-introduction group but were distinguished from post-vaccination observations collected at least 12 months after national vaccine introduction.

Studies were excluded if they involved populations older than five years without providing separate data for children under five, focused on animal subjects, or were designed as case reports, editorials, commentaries, or review articles. Publications that did not report genotype data specific to Central Asia were excluded, as were duplicate reports of the same dataset. Only studies published in English were considered for inclusion. The language restriction was applied because of resource limitations and the need to ensure consistent assessment of study eligibility and data extraction. However, relevant surveillance studies may have been published in Russian or other regional languages, which could have resulted in under-ascertainment of eligible studies.

### 2.3. Selection of Studies and Data Extraction

PRISMA guidelines were followed for the study selection and data extraction phases. Duplicate records were removed manually based on title, authors, publication year, and journal information. Afterwards, two independent reviewers (Y.S. and I.K.) evaluated the eligibility of the chosen studies based on their titles and abstracts. Full-text articles of relevant studies deemed worthy of inclusion were retrieved and assessed. The following data were extracted: first author’s surname, year of publication, country of study, study design, surveillance site and location, total sample size, number of rotavirus-positive cases used as the denominator for genotype analysis, exact data collection dates, diagnostic assay, genotyping method, genotype distribution (G1P[8], G2P[4], G3P[8], G4P[8], G9P[8], G9P[4], G12P[8], mixed genotypes, and non-typeable strains), vaccination status, vaccine product, national vaccine coverage at the time of data collection, interval since national vaccine introduction, and potential overlap with other included reports.

Two reviewers (Y.S. and I.K.) independently conducted data extraction using a standardized data collection form. Any discrepancies in data extraction were resolved through discussion or consultation with a third reviewer (Sh.S.). No data were calculated or imputed other than those specifically stated in the original studies.

### 2.4. Risk of Bias (Quality) Assessment

Risk of bias was independently assessed by two reviewers (YS and IK) using the Joanna Briggs Institute (JBI) Critical Appraisal Checklist for Studies Reporting Prevalence Data [16]. Judgments were recorded for each checklist item as Yes, No, Unclear, or Not Applicable. Disagreements were resolved through discussion and, when necessary, consultation with a third reviewer until consensus was reached. An overall summary of the risk of bias assessment is provided in Table 1. No numerical quality score or unvalidated threshold was used to exclude studies.

### 2.5. Certainty of Evidence Assessment

The overall certainty of evidence for genotype-specific pooled prevalence estimates was evaluated using the Grading of Recommendations Assessment, Development and Evaluation (GRADE) framework. The assessment considered five domains: risk of bias, inconsistency, indirectness, imprecision, and publication bias. Evidence from observational studies was initially rated as low certainty and subsequently downgraded or upgraded based on domain-specific criteria. Consistent with standard GRADE practice (Cochrane Handbook, Chapter 14 [13]), observational designs begin at low certainty by default—reflecting greater susceptibility to confounding and selection bias than randomized designs, which begin at high certainty—and the five GRADE domains were applied analogously to prevalence outcomes (risk of bias, inconsistency, indirectness, imprecision, publication bias) rather than to an intervention effect. Inconsistency was primarily assessed using between-study heterogeneity (I^2^ statistics), while imprecision was evaluated based on the width of confidence intervals and sample size. Because fewer than 10 independent studies contributed to each genotype-specific pooled analysis, publication bias was not formally assessed. Outcome-specific certainty was downgraded for serious risk of bias, substantial inconsistency (high between-study heterogeneity), indirectness (ecological pre-/post-vaccination comparisons and differences in surveillance systems), and imprecision (small numbers of studies or wide confidence intervals), where applicable. The final certainty of evidence for each outcome was categorized as high, moderate, low, or very low.

### 2.6. Statistical Analysis Plan

The primary unit of analysis was the independent study-period. Country-year observations derived from the same surveillance dataset were aggregated where appropriate, and overlapping reports were not included simultaneously. Genotype-specific proportions were calculated using the number of samples tested for the relevant genotype as the denominator; genotype categories not reported by a study were treated as missing rather than as zero events. Random-effects meta-analysis of proportions was conducted using a random-intercept logistic-normal generalized linear mixed model with a logit link. Between-study heterogeneity was summarized using tau-squared and I-squared, and pooled estimates were reported with 95% confidence intervals. Subgroup comparisons were limited to the before and after vaccine introduction groups, with transition-period observations retained within the latter. Additional quantitative subgroup analyses were not performed because of the small number of independent studies. Tests for funnel-plot asymmetry, including Egger regression, were not conducted because fewer than 10 independent studies contributed to each outcome. Analyses were performed in R (version 4.4.1) using the meta and metafor packages (R Foundation for Statistical Computing, Vienna, Austria) [22,23,24].

## 3. Results

### 3.1. Study Selection

**Figure 1:**
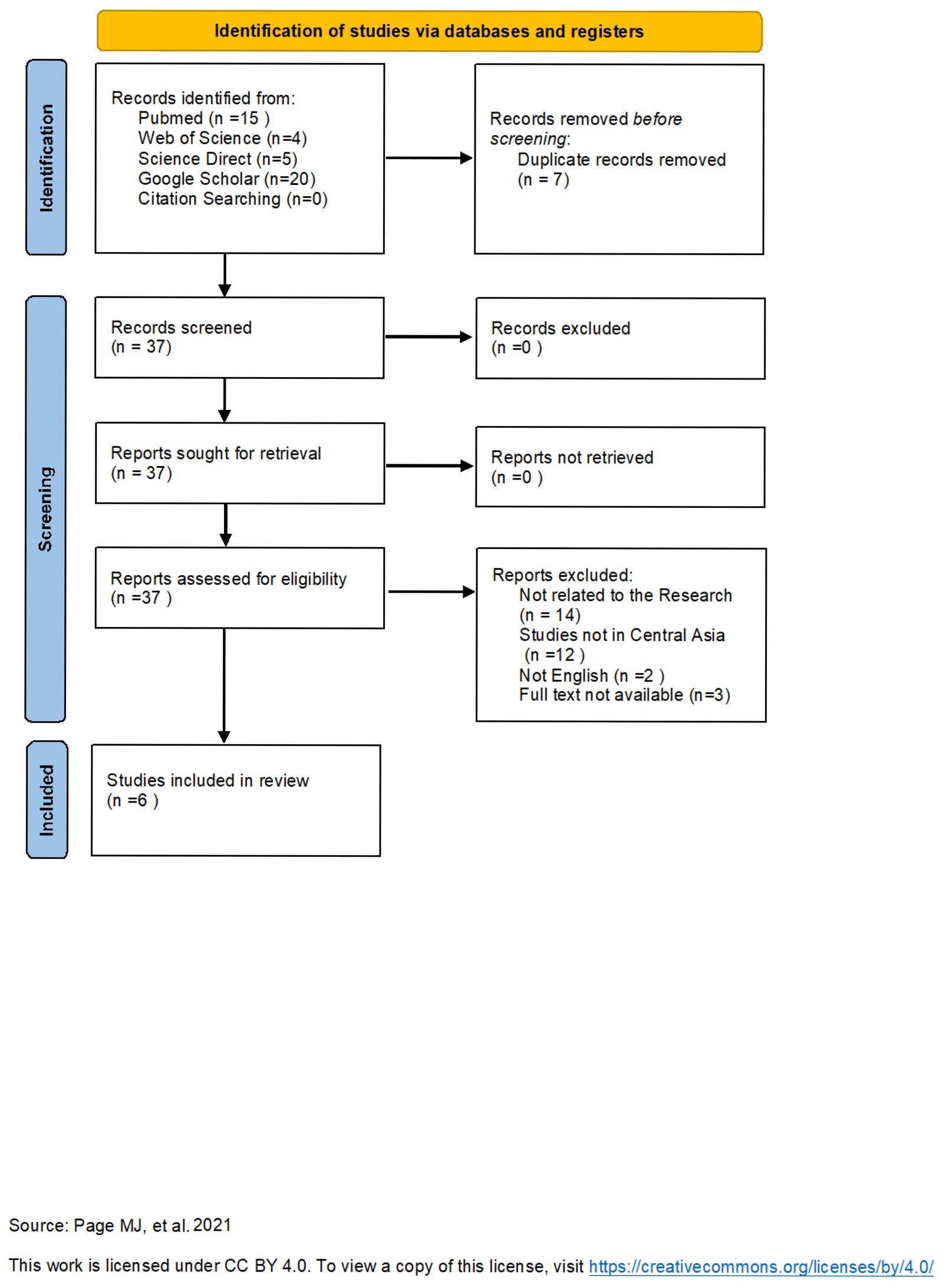
PRISMA 2020 flow diagram for study selection [15].

The PRISMA flow diagram of the study selection process is presented in Figure 1. A total of 44 records were identified through database searches, including Web of Science (*n* = 4), PubMed (*n* = 15), ScienceDirect (*n* = 5), and Google Scholar (*n* = 20). After removal of 7 duplicate records, 37 records remained for screening. During study screening and full-text eligibility assessment, 31 reports were excluded because they were not related to the research question (*n* = 14), were conducted outside Central Asia (*n* = 12), were not published in English (*n* = 2), or the full text was unavailable (*n* = 3). Consequently, six studies met the inclusion criteria and were included in the systematic review and meta-analysis. Several studies were excluded for not being directly related to the research question, including Latipov et al. [5], de Blasio et al. [11], and Musabaev et al. [25].

### 3.2. Characteristics of Included Studies

The included studies covered several countries in Central Asia and were published between 2009 and 2021. The majority of studies included children under five years of age hospitalized with acute gastroenteritis or acute diarrhea. The included investigations consisted of prospective and observational surveillance studies conducted during pre-vaccination, transition, and post-vaccination periods. The studies varied in surveillance settings, geographic location, laboratory methods, and vaccination status, reflecting regional differences in rotavirus epidemiology. The characteristics of the included studies and study periods are summarized in Table 2.

### 3.3. Pooled Prevalence of Rotavirus Genotypes

**Figure 2:**
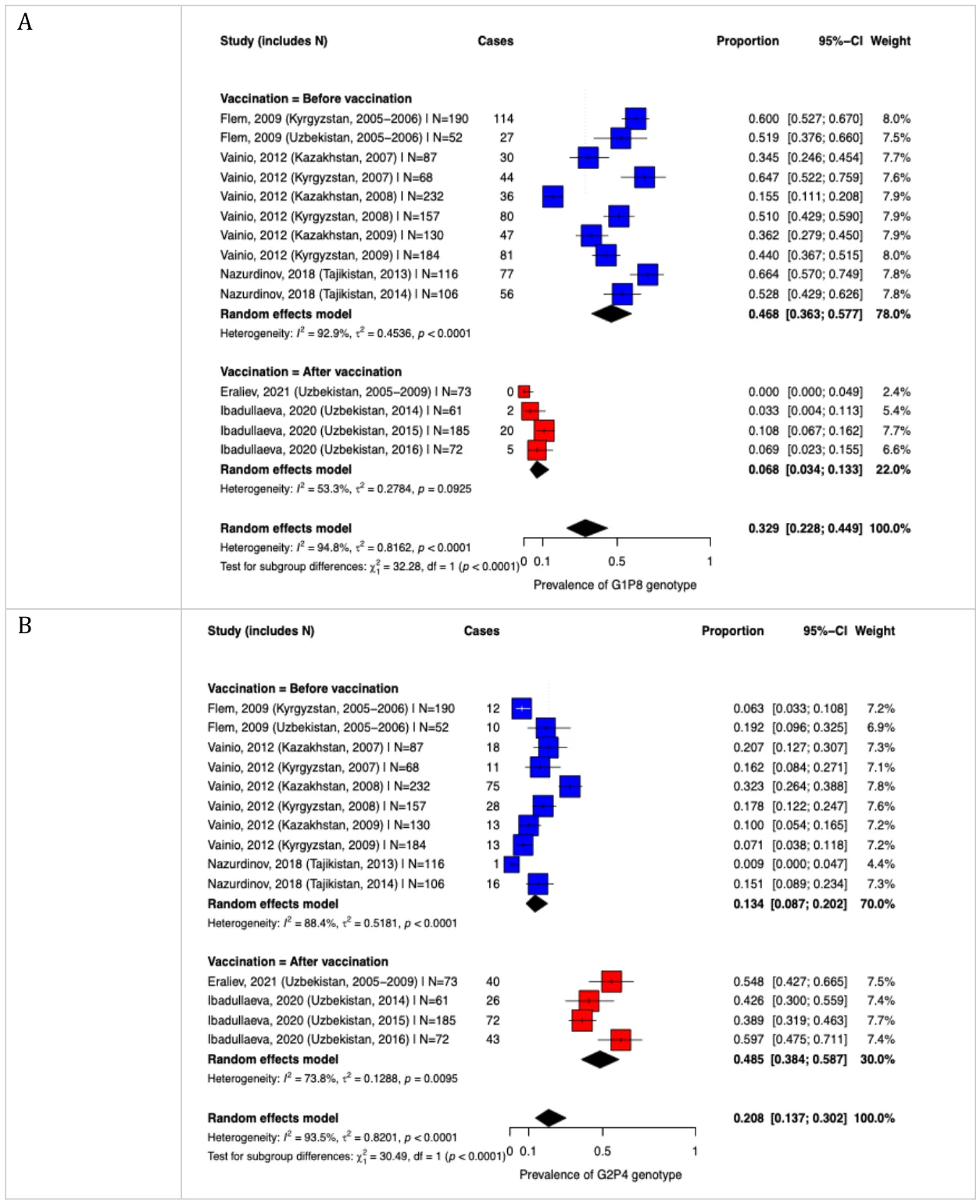

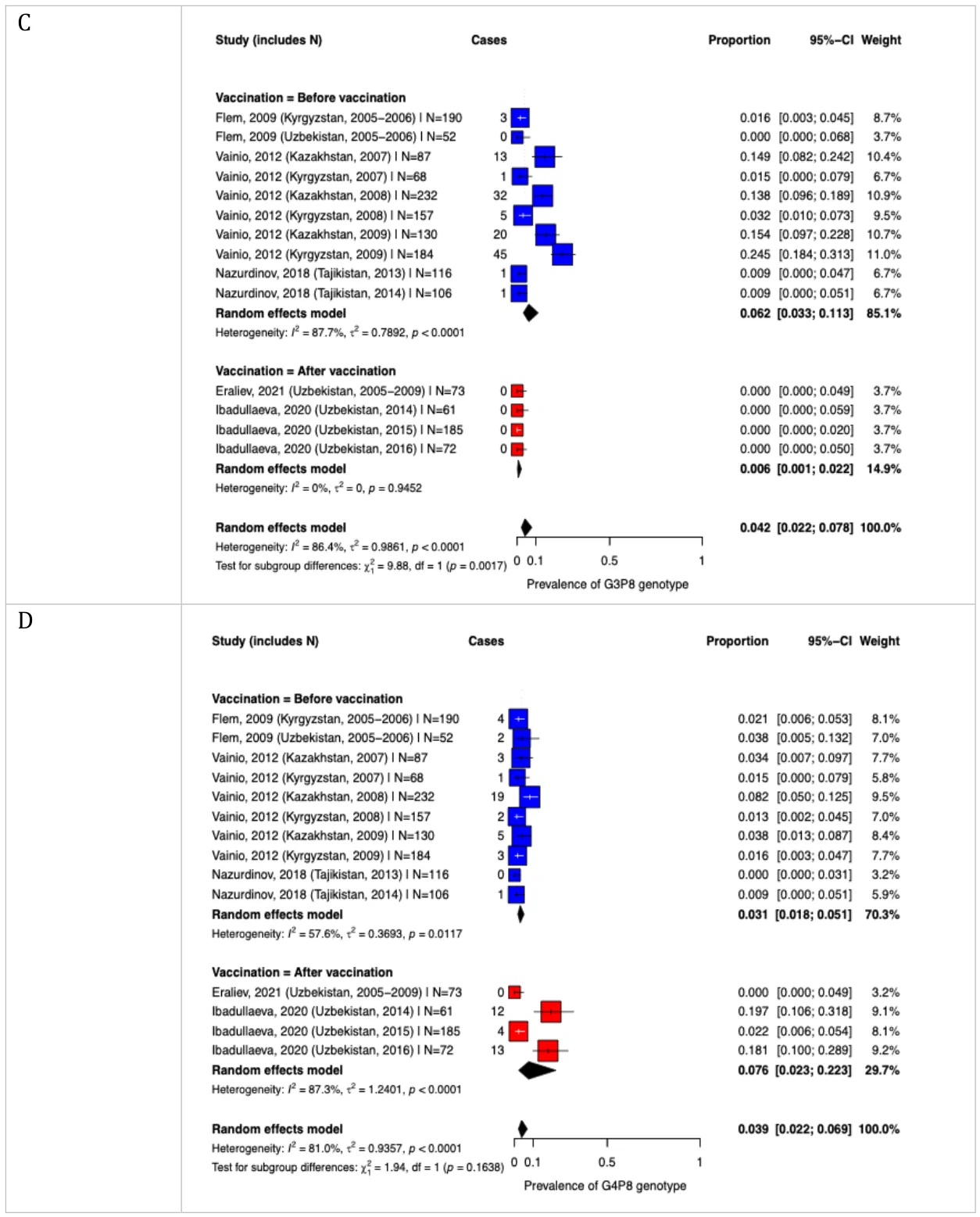

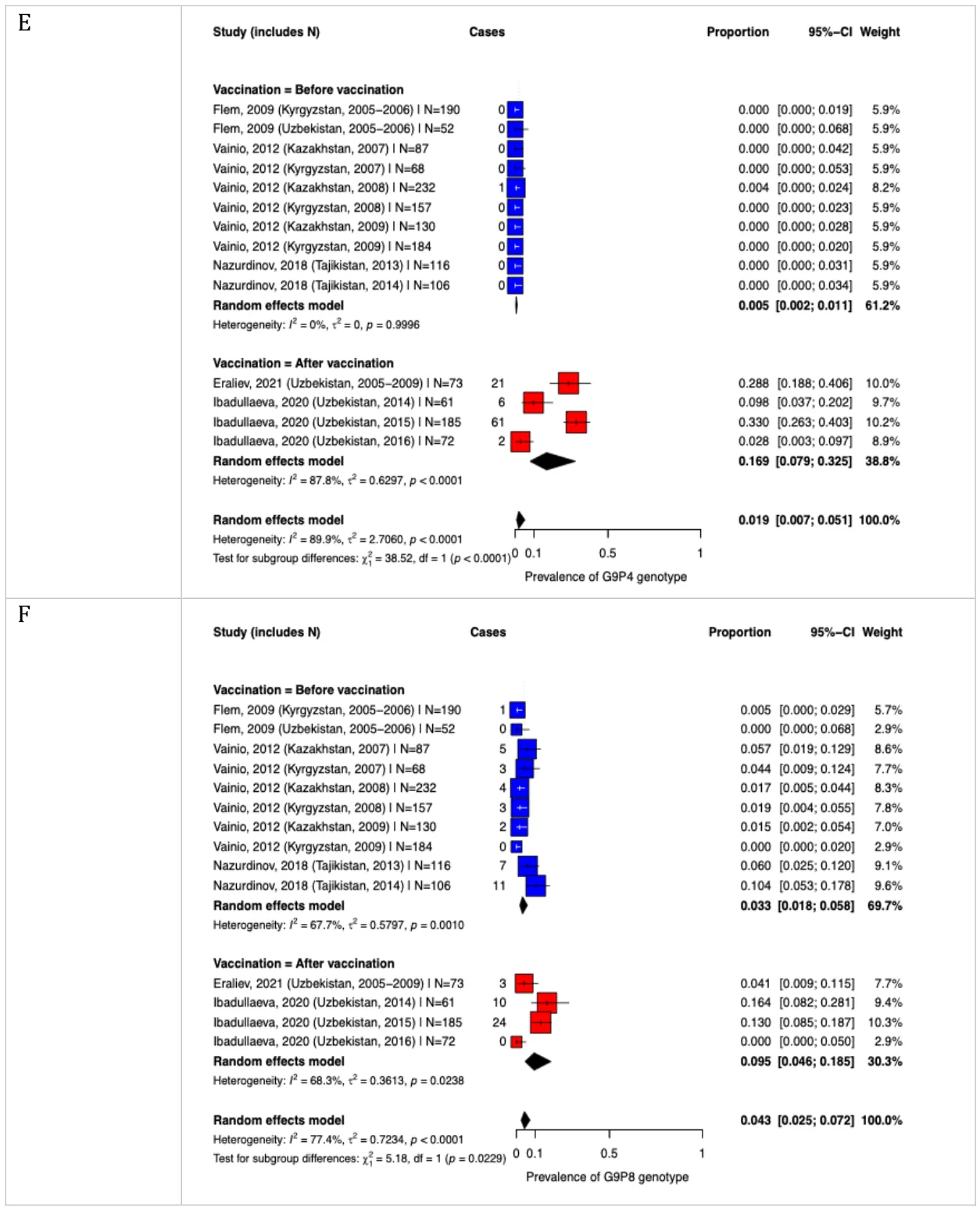

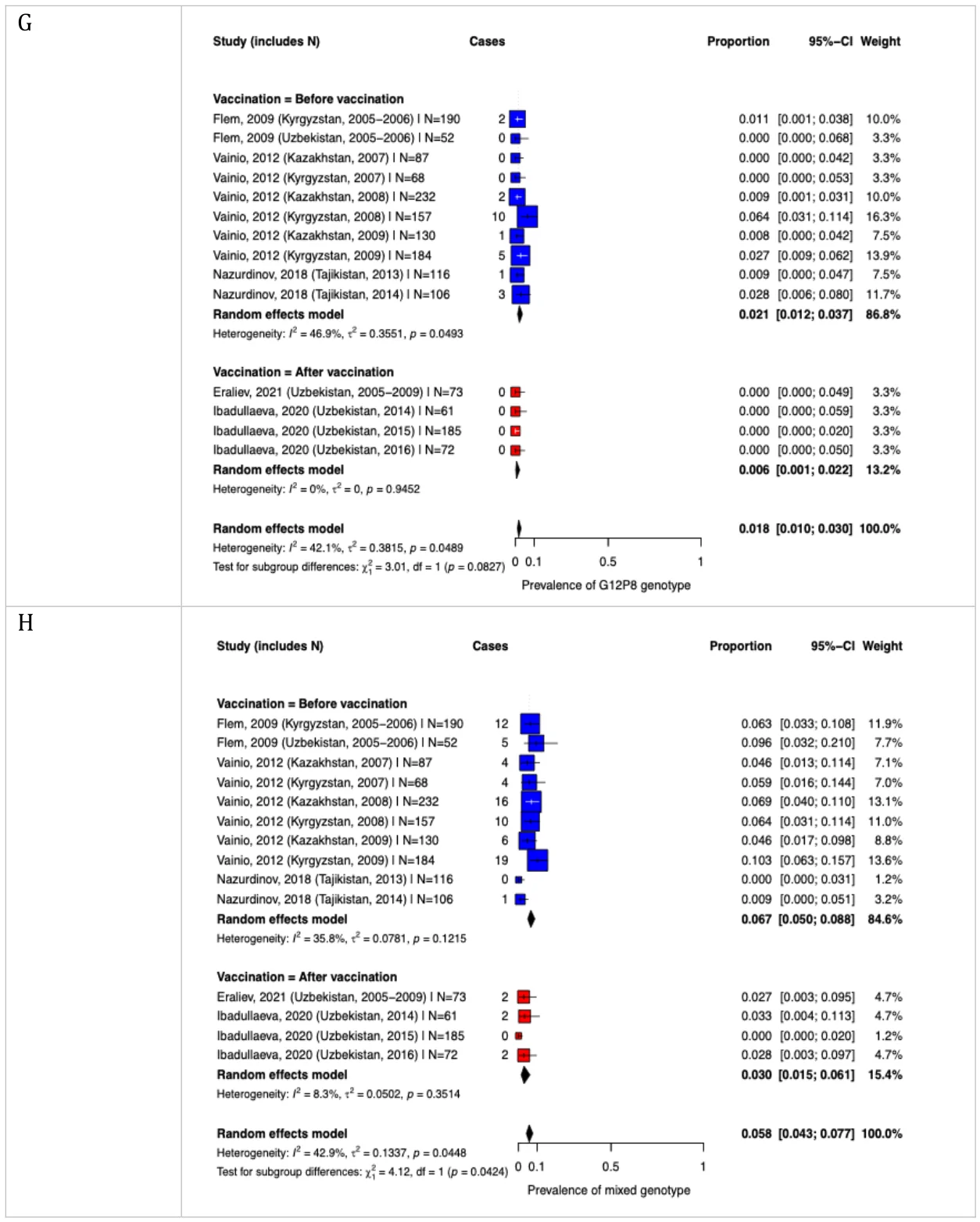

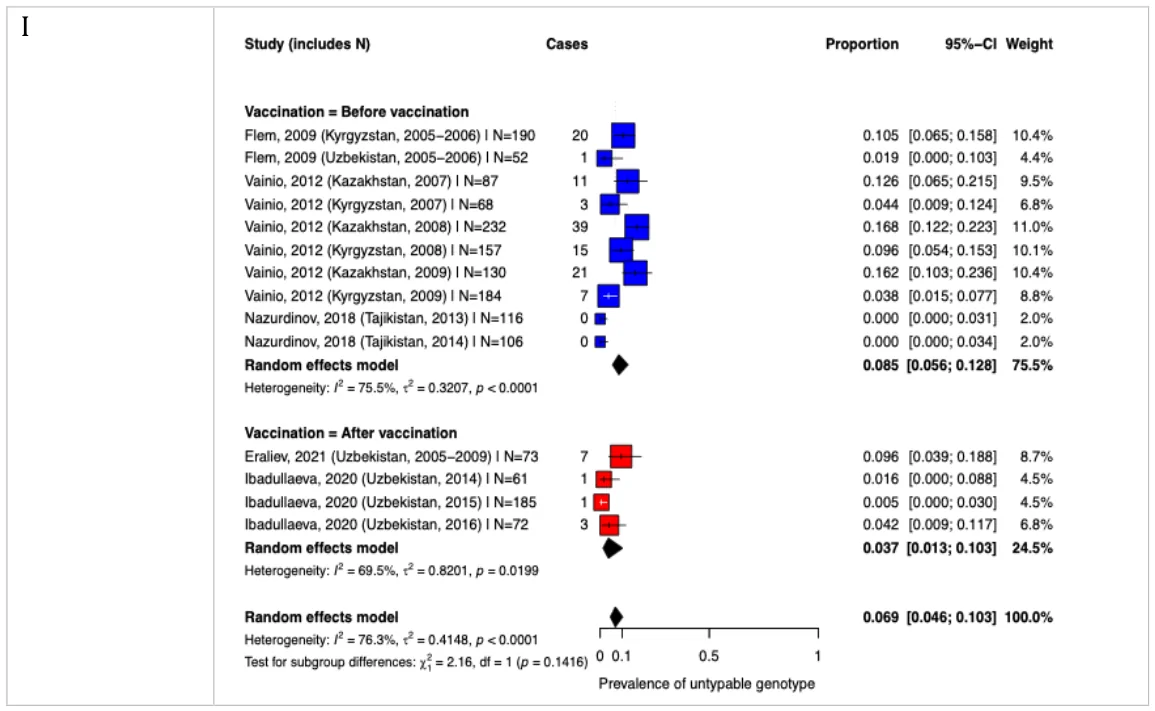
Forest plots of pooled prevalence of rotavirus genotypes before and after vaccine introduction. The after vaccine introduction group included both transition-period observations collected during the first 12 months after vaccine introduction and observations collected at least 12 months after vaccine introduction. (**A**) G1P[8]; (**B**) G2P[4]; (**C**) G3P[8]; (**D**) G4P[8]; (**E**) G9P[4]; (**F**) G9P[8]; (**G**) G12P[6]; (**H**) Mixed genotype; (**I**) Untypable genotype.

The pooled proportions of rotavirus genotypes among genotyped rotavirus-positive samples before and after vaccine introduction are presented in Figure 2A–I. Random-effects models were applied because of substantial between-study heterogeneity.

A lower pooled proportion of G1P[8] was observed in before vaccine introduction strata than in post vaccine introduction strata (Figure 2A). The pooled prevalence was 0.329 (95% CI: 0.228–0.449; $I^{2}=94.8\%$). Before vaccine introduction prevalence was 0.468 (95% CI: 0.363–0.577), decreasing to 0.068 (95% CI: 0.034–0.133) after vaccine introduction ($\chi^{2}=32.28$, p<0.0001). Higher pooled proportions of G2P[4] were observed in after vaccine introduction strata than in before vaccine introduction strata (Figure 2B). The pooled prevalence was 0.208 (95% CI: 0.137–0.302; $I^{2}=93.5\%$), increasing from 0.134 pre-vaccination to 0.485 post-vaccination ($\chi^{2}=30.49$, p<0.0001). Given I^2^ values exceeding 90% for both G1P[8] and G2P[4], these pooled proportions should be interpreted as descriptive summaries of highly variable underlying data rather than precise estimates of a single regional prevalence; subgrouping by vaccination status explains only part of this variability, and substantial heterogeneity remains within each subgroup.

G3P[8] declined substantially in after vaccine introduction strata (Figure 2C), with prevalence decreasing from 0.062 to 0.006 ($\chi^{2}=9.88$, p=0.0017).

G4P[8] showed no statistically significant subgroup difference (Figure 2D; p=0.1638). Pronounced increases were observed for G9P[4] (Figure 2E) and G9P[8] (Figure 2F) after vaccine introduction strata.

G12P[8] remained relatively stable, whereas mixed genotypes showed a modest but statistically significant decline following vaccination. Untypable strains also decreased in after vaccine introduction strata, although subgroup differences were not statistically significant.

The genotype-specific evidence contributing to the pre-vaccination and post-vaccination subgroup analyses is summarized in Table 3. Across all genotype outcomes, 10 pre-vaccination strata involving 1322 rotavirus-positive children and four post-vaccination strata involving 391 children contributed to the analyses, although the numbers of genotype-positive observations varied substantially between outcomes.

### 3.4. Sensitivity Analysis

Influence diagnostics did not identify a single consistently dominant stratum; however, the small number of independent studies limits the interpretability of these diagnostics.

The certainty of evidence for the ecological differences between before and after vaccine introduction surveillance periods was rated as very low for all nine genotype outcomes (Table 4). The evidence base comprised six included publications overall, contributing 10 before vaccine introduction strata involving 1322 rotavirus-positive children and four after vaccine introduction strata involving 391 children. The number of genotype-positive observations varied substantially, ranging from one before vaccine introduction G9P[4] observation and no after vaccine introduction G3P[8] or G12P[8] observations to 592 before vaccine introduction G1P[8] and 181 after vaccine introduction G2P[4] observations. Although the JBI appraisal did not identify serious within-study risk-of-bias concerns, all outcomes were downgraded for serious indirectness. Vaccination status was assigned ecologically according to country and surveillance period rather than individual vaccination history, and all after vaccine introduction observations came from Uzbekistan, whereas the before vaccine introduction group also included data from Kazakhstan, Kyrgyzstan and Tajikistan. The comparison was therefore confounded by calendar period, country representation, surveillance setting, vaccine coverage, participant selection and laboratory methods. Substantial residual heterogeneity resulted in further downgrading for G1P[8], G2P[4], G3P[8], G4P[8], G9P[4], G9P[8] and untypable strains. Evidence for G3P[8], G4P[8], G9P[8], G12P[8], mixed genotypes and untypable strains was also limited by imprecision because of sparse after vaccine introduction events and/or wide confidence intervals. Publication bias could not be assessed reliably because fewer than 10 independent studies contributed, and selective reporting could not be excluded. No upgrading was applied because the observed subgroup differences represent ecological temporal contrasts and cannot establish a causal vaccine effect. These ratings support interpreting the genotype findings as hypothesis-generating rather than confirmatory.

## 4. Discussion

### 4.1. Principal Findings

This review identified a small and uneven evidence base on rotavirus genotype distribution among children younger than five years in Central Asia. In the current pooled analysis, post-introduction strata had a lower proportion of G1P[8] and higher proportions of G2P[4] and selected G9 genotypes than pre-introduction strata, while G3P[8] was less frequent. These findings describe differences between surveillance periods rather than a vaccine-effect estimate. The post-introduction evidence came exclusively from Uzbekistan, whereas Kazakhstan and the included Kyrgyz and Tajik data contributed only pre-introduction observations. The regional interpretation is therefore limited, and the estimates should be regarded as hypothesis-generating.

### 4.2. Interpretation of Genotype Patterns

The lower proportion of G1P[8] is biologically compatible with changes following the introduction of a monovalent G1P[8] vaccine and with the documented heterotypic protection of rotavirus vaccines against some partially and fully heterotypic strains [27,28,29]. However, genotype proportions alone cannot establish that vaccination caused the observed change. Rotavirus genotype distributions fluctuate naturally across countries and calendar periods, and a reduction in the relative proportion of one genotype necessarily increases the proportional contribution of others even when their absolute incidence does not increase [10,27,30,31]. Recent global analyses found that crude comparisons may suggest higher G2P[4] prevalence in vaccine-introducing settings, but this association is substantially attenuated after accounting for temporal and regional patterns, providing little evidence of sustained vaccine-driven selective pressure [27]. The present review did not estimate genotype-specific hospitalization incidence, population incidence, absolute numbers of infections, or strain-specific vaccine effectiveness. Consequently, terms such as vaccine-driven strain replacement, vaccine escape, successful interruption of transmission, or vaccine failure are not supported by these data. The results are more appropriately interpreted as temporal differences in the relative composition of genotyped rotavirus-positive samples.

### 4.3. Competing Explanations and Heterogeneity

The higher proportions of G2P[4] and G9-associated genotypes in the post-introduction strata are consistent with patterns reported in some post-licensure settings, although natural genotype cycling remains an equally plausible explanation [27,30,32,33]. Recent surveillance and systematic reviews have demonstrated considerable geographic and temporal genotype diversity, without identifying a consistently dominant post-vaccine genotype or convincing evidence of long-term vaccine-driven strain displacement [10,27,30]. The pre- and post-introduction observations differed simultaneously by calendar period, country representation, vaccine coverage, surveillance design, participant selection, sampling strategy, and laboratory methods [4,17,18,19,20,21]. Earlier and later studies did not necessarily use identical antigen-detection assays, genotyping primers, selection fractions, or definitions of typeable, mixed, and untypeable strains. These methodological factors are partly confounded with vaccination status and cannot be separated statistically in a dataset containing only a few independent studies. Differences in laboratory sensitivity and genotype assignment may therefore have contributed to the observed between-period contrasts. The very high heterogeneity for major genotypes reinforces this limitation. Under I^2^ values exceeding 90%, pooled proportions summarize markedly disparate surveillance estimates and should not be interpreted as a single underlying regional prevalence. Vaccination-status subgrouping explained only part of this variability, and residual heterogeneity remained substantial. The pooled values are therefore descriptive averages rather than precise estimates applicable to all Central Asian countries.

### 4.4. Vaccinating and Non-Vaccinating Settings

All post-introduction genotype observations included in this review were from Uzbekistan [20,21]. These results should not be extrapolated directly to Kazakhstan or to Central Asia as a whole. Kazakhstan contributed historical pre-vaccine surveillance data only, and no included study provided a within-country before-and-after genotype comparison for Kazakhstan [18]. Similarly, the review identified no eligible post-introduction genotype series from Kyrgyzstan, Tajikistan, or Turkmenistan. Evidence from Uzbekistan indicates that the national monovalent vaccination programme reduced rotavirus-positive and rotavirus-associated hospitalizations among young children [3,34]. However, evidence that vaccination reduced the burden of severe disease should be distinguished from the present review’s ecological comparison of genotype proportions. The Uzbekistan impact studies support the effectiveness of the vaccination programme against rotavirus disease, but they do not demonstrate that vaccination was the sole cause of the observed genotype differences. A more informative regional assessment will require paired, country-specific surveillance using comparable sentinel sites, case definitions, sampling procedures, and laboratory protocols before and after vaccine introduction [27,33,35]. Until such evidence is available, country-level patterns should be presented separately and regional pooled estimates interpreted cautiously.

### 4.5. Public Health Implications

The findings support strengthening molecular surveillance, but they should not be used as stand-alone evidence either for or against rotavirus vaccine introduction. National vaccination decisions should primarily be based on rotavirus disease burden, vaccine effectiveness and safety, programme feasibility, affordability, cost-effectiveness, achievable coverage, and equitable access [11,16,21,28,36,37]. Reviews of economic evaluations generally support the cost-effectiveness of rotavirus vaccination in low- and lower-middle-income settings, while also emphasizing the need for reliable country-specific incidence, cost, vaccine-price, and programme-delivery data [37]. Genotype surveillance has a complementary role. It can identify sustained changes in circulating strains, monitor the proportion of mixed and untypeable samples, detect unusual reassortants, and determine whether genotype changes coincide with changes in disease severity or vaccine performance [10,31,35]. Recent surveillance experience supports the use of coordinated laboratory networks and increasingly emphasizes whole-genome sequencing to characterize reassortment and genetic diversity beyond conventional G- and P-typing [10]. A practical Central Asian surveillance framework should apply common case definitions, record exact vaccination status and age eligibility, report the number of children screened, tested, rotavirus-positive, and successfully genotyped, and use standardized RT-PCR or sequencing protocols. Genotype data should be linked to hospitalization severity, vaccination history, geographic location, and population denominators. Annual reporting of vaccine product, dose-specific coverage, and time since national introduction would permit future analyses to distinguish early transition periods from mature vaccination programmes [10,27,35].

### 4.6. Strengths and Limitations

This review addresses an underrepresented region, uses duplicate screening and data extraction, and presents genotype-specific quantitative summaries. Its conclusions are nevertheless constrained by the small number of independent studies, the concentration of post-introduction data in one country, possible overlap between reports derived from related surveillance platforms, repeated country-year observations within studies, sparse data for uncommon genotypes, the English-language restriction, and substantial clinical and methodological heterogeneity. The possibility of overlapping surveillance populations is particularly important because treating non-independent observations as separate strata may produce overly narrow confidence intervals and give disproportionate weight to individual surveillance systems. Publication bias could not be evaluated reliably because too few independent studies were available for adequately powered funnel-plot asymmetry testing. Selective publication or selective genotype reporting therefore cannot be excluded. In addition, some articles did not report every genotype category consistently; an unreported genotype should not be assumed to represent zero cases unless this is explicitly supported by the original report. The classification of the early post-introduction period also requires caution. The 2014 Uzbekistan surveillance period was classified as a transition period data and excluded from the primary pre-/post-vaccination comparison; however, we acknowledge that it represented an early transitional phase following vaccine introduction, during which vaccine coverage and population-level effects may not yet have been fully established [20,27]. The Uzbekistan data collected during October–December 2014 represented a transition period within the first year after vaccine introduction. Although these observations were retained in the primary after-introduction analysis, their inclusion may have attenuated the estimated difference between the pre-introduction and established post-vaccination periods.

Overall, the available evidence suggests temporal differences in rotavirus genotype composition in Uzbekistan after vaccine introduction, but it does not demonstrate causal vaccine-driven strain replacement and does not establish a regional post-vaccination pattern. Coordinated, longitudinal, multicountry molecular surveillance is needed to determine whether the observed differences persist, reflect natural genotype cycling, or are associated with vaccination and concurrent changes in rotavirus disease burden [10,27,31,35].

## 5. Conclusions

The available evidence suggests differences in rotavirus genotype composition between pre-introduction and post-introduction surveillance periods, including a lower proportion of G1P[8] and higher proportions of G2P[4] and selected G9-associated genotypes in post-introduction observations. However, the post-introduction evidence is limited to Uzbekistan, the number of independent datasets is small, and the comparison is confounded by calendar time, study setting, laboratory methods, vaccine coverage, the early transitional status of the 2014 surveillance period, and possible overlap between reports. These findings should therefore be considered hypothesis-generating rather than evidence of causal vaccine-driven strain replacement. Standardized, longitudinal, multicountry molecular surveillance linked to individual vaccination status, disease severity, and population-level disease-burden data is required before regional conclusions can be drawn.

## Figures and Tables

**Table 1:** Risk of bias assessment using the Joanna Briggs Institute (JBI) Critical Appraisal Checklist for Studies Reporting Prevalence Data.

Study	Q1	Q2	Q3	Q4	Q5	Q6	Q7	Q8	Q9	Overall
Flem et al., 2009 [17]	Y	Y	Y	Y	Y	Y	Y	Y	Y	Low
Flem et al., 2009 [4]	Y	Y	Y	Y	Y	Y	Y	Y	Y	Low
Vainio et al., 2013 [18]	Y	Y	Y	Y	Y	Y	Y	Y	Y	Low
Nazurdinov et al., 2017 [19]	Y	Y	Y	Y	Y	Y	Y	Y	Y	Low
Ibadullaeva et al., 2020 [20]	Y	Y	Y	Y	Y	Y	Y	Y	Y	Low
Eraliev et al., 2021 [21]	Y	Y	Y	Y	Y	Y	Y	Y	Y	Low

**Abbreviations:** JBI, Joanna Briggs Institute; Y, Yes. Q1, Was the sample frame appropriate to address the target population?; Q2, Were study participants sampled appropriately?; Q3, Was the sample size adequate?; Q4, Were the study subjects and setting described in detail?; Q5, Was the data analysis conducted with sufficient coverage of the identified sample?; Q6, Were valid methods used for the identification of the condition?; Q7, Was the condition measured in a standard, reliable way for all participants?; Q8, Was appropriate statistical analysis used?; Q9, Was the response rate adequate, and if not, was the low response rate managed appropriately?

**Table 2:** Characteristics of the included studies and analytic periods.

Study	Design and Population	Country and Surveillance Sites	Data Collection Period and Analytic Subgroup	Vaccine Information	Laboratory Methods
Flem et al., 2009 [17]	Prospective hospital-based sentinel surveillance of children aged <5 years hospitalized with acute gastroenteritis	Kyrgyzstan: Republican Hospital of Infectious Diseases, Bishkek; Children’s Hospital and County Hospital, Osh	2005–2006; pre-vaccination	No national rotavirus vaccination programme during data collection; coverage not applicable; before national introduction	DAKO IDEIA rotavirus ELISA; G- and P-genotyping by RT-PCR at reference laboratories
Flem et al., 2009 [4]	Observational hospital-based sentinel surveillance of children aged <5 years hospitalized with acute gastroenteritis	Uzbekistan: Pediatric Infectious Disease Hospital No. 4, Tashkent; Regional Infectious Disease Hospital, Bukhara	1 January 2005–31 December 2006; pre-vaccination	No national rotavirus vaccination programme during data collection; coverage not applicable; before national introduction	IDEIA Rotavirus ELISA; G- and P-genotyping by RT-PCR at the US CDC
Vainio et al., 2013 [18]	Prospective hospital-based sentinel surveillance of children aged <5 years admitted with acute gastroenteritis	Kyrgyzstan: Republican Hospital of Infectious Diseases, Bishkek, and Children’s Hospital, Osh; Kazakhstan: Pediatric Infectious Diseases Hospital, Almaty, and Regional Infectious Disease Hospital, Karaganda	January 2007–December 2009; country-year observations contributed to the pre-vaccination subgroup	No national rotavirus vaccination programme during data collection; coverage not applicable; before national introduction	IDEIA or RIDASCREEN rotavirus ELISA; semi-nested G- and P-typing RT-PCR; sequencing of selected strains
Nazurdinov et al., 2017 [19]	Prospective sentinel surveillance of children aged <5 years hospitalized with acute gastroenteritis	Tajikistan: Children’s Clinical Infectious Disease Hospital and Adult Infectious Disease Hospital, Dushanbe	January 2013–December 2014; pre-vaccination	No national rotavirus vaccination programme during data collection; coverage not applicable; before national introduction	ProSpecT Rotavirus ELISA; PCR genotyping at the WHO Regional Reference Laboratory, Minsk
Ibadullaeva et al., 2020 [20]	Cross-sectional laboratory surveillance of children aged <5 years hospitalized with acute gastroenteritis	Uzbekistan: City Infectious Diseases Hospital No. 4, Tashkent; Regional Infectious Diseases Hospital, Bukhara	October–December 2014: transition period; included in the primary analysis within the after-introduction group. 2015–2016: post-vaccination period, included in the primary analysis	Rotarix; estimated national coverage was 52% in 2014, 95% in 2015, and 99% in 2016. The 2014 period began <12 months after introduction; the 2015–2016 period occurred 1–2 years after introduction	ProSpecT Rotavirus EIA; one-step conventional RT-PCR using CDC genotyping primers
Eraliev et al., 2021 [21]	Sentinel surveillance with a test-negative case-control component among children aged <5 years hospitalized with acute diarrhea	Uzbekistan: Fourth Tashkent City Infectious Diseases Hospital, Tashkent; Bukhara Regional Infectious Diseases Hospital, Bukhara	2015–2016; post-vaccination only	Rotarix; estimated national coverage was 95% in 2015 and 99% in 2016; surveillance occurred 1–2 years after national introduction	ProSpecT Rotavirus EIA; genotyping by RT-PCR at the US CDC

Note: CDC, Centers for Disease Control and Prevention; EIA, enzyme immunoassay; ELISA, enzyme-linked immunosorbent assay; RT-PCR, reverse transcription polymerase chain reaction. For the primary subgroup analysis, a country-period was classified as post-vaccination only when data collection began at least 12 months after national vaccine introduction. Data collected during the first 12 months after introduction were classified as transition-period observations and retained in the primary analysis within the broader after-vaccine-introduction group. Vaccine introduction dates and national coverage estimates were obtained from the WHO Immunization Data Portal and the WHO/UNICEF Estimates of National Immunization Coverage (WUENIC) [10,26]. Potential overlap between surveillance reports was assessed using country, surveillance site, study period, participant population, and laboratory methods. Overlapping datasets were not included simultaneously in the quantitative synthesis. Only post-vaccination genotype data from Eraliev et al. [21] were included in the present analysis.

**Table 3:** Genotype-specific evidence by vaccination subgroup.

Genotype	*k* (Pre-Vacc.)	*N* (Pre-Vacc.)	Cases (Pre-Vacc.)	*k* (Post-Vacc.)	*N* (Post-Vacc.)	Cases (Post-Vacc.)
G1P[8]	10	1322	592	4	391	27
G2P[4]	10	1322	197	4	391	181
G3P[8]	10	1322	121	4	391	0
G4P[8]	10	1322	40	4	391	29
G9P[4]	10	1322	1	4	391	90
G9P[8]	10	1322	36	4	391	37
G12P[8]	10	1322	24	4	391	0
Mixed	10	1322	77	4	391	6
Untypable	10	1322	117	4	391	12

Note: *k* indicates the number of contributing study strata; *N* indicates the total number of rotavirus-positive children included in the genotype analysis; Cases indicates the number of genotype-positive observations.

**Table 4:** Certainty of evidence for ecological differences in rotavirus genotype proportions between before and after vaccine introduction surveillance periods.

Genotype	Before Vaccination Evidence: Publications/Strata; *N*; Cases; Pooled Proportion (95% CI)	After Vaccine Introduction Evidence: Publications/Strata; *N*; Cases; Pooled Proportion (95% CI)	Subgroup *p*-Value	Risk ofBias ^1^	Inconsistency ^2^	Indirectness ^3^	Imprecision ^4^	PublicationBias ^5^	Certainty ^6^
G1P[8]	5/10; 1322; 592; 0.468 (0.363–0.577)	2/4; 391; 27; 0.068 (0.034–0.133)	<0.0001	Not serious	Serious	Serious	Not serious	Not assessable	Very low ⊕◯◯◯
G2P[4]	5/10; 1322; 197; 0.134 (0.087–0.202)	2/4; 391; 181; 0.485 (0.384–0.587)	<0.0001	Not serious	Serious	Serious	Not serious	Not assessable	Very low ⊕◯◯◯
G3P[8]	5/10; 1322; 121; 0.062 (0.033–0.113)	2/4; 391; 0; 0.006 (0.001–0.029)	0.0017	Not serious	Serious	Serious	Serious	Not assessable	Very low ⊕◯◯◯
G4P[8]	5/10; 1322; 40; 0.031 (0.018–0.051)	2/4; 391; 29; 0.076 (0.023–0.232)	0.1638	Not serious	Serious	Serious	Serious	Not assessable	Very low ⊕◯◯◯
G9P[4]	5/10; 1322; 1; 0.005 (0.002–0.011)	2/4; 391; 90; 0.169 (0.079–0.325)	<0.0001	Not serious	Serious	Serious	Not serious	Not assessable	Very low ⊕◯◯◯
G9P[8]	5/10; 1322; 36; 0.033 (0.018–0.058)	2/4; 391; 37; 0.095 (0.046–0.185)	0.0229	Not serious	Serious	Serious	Serious	Not assessable	Very low ⊕◯◯◯
G12P[8]	5/10; 1322; 24; 0.021 (0.012–0.037)	2/4; 391; 0; 0.006 (0.001–0.029)	0.0827	Not serious	Not serious	Serious	Serious	Not assessable	Very low ⊕◯◯◯
Mixed genotypes	5/10; 1322; 77; 0.067 (0.050–0.088)	2/4; 391; 6; 0.030 (0.015–0.061)	0.0424	Not serious	Not serious	Serious	Serious	Not assessable	Very low ⊕◯◯◯
Untypable strains	5/10; 1322; 117; 0.085 (0.056–0.128)	2/4; 391; 12; 0.037 (0.013–0.103)	0.1416	Not serious	Serious	Serious	Serious	Not assessable	Very low ⊕◯◯◯

Notes: ^1^ Risk of bias was assessed using the Joanna Briggs Institute Critical Appraisal Checklist for Studies Reporting Prevalence Data. ^2^ Inconsistency was downgraded when substantial residual heterogeneity remained. ^3^ Indirectness was downgraded because vaccination status was assigned ecologically by country and surveillance period rather than individual vaccination history, and all observations after vaccine introduction originated from Uzbekistan. ^4^ Imprecision was downgraded for sparse events and/or wide confidence intervals. ^5^ Publication bias was not assessable because fewer than 10 independent studies contributed; selective reporting could not be excluded. ^6^ Certainty was rated using an adapted GRADE framework for ecological differences in genotype proportions. GRADE certainty ratings: ⊕◯◯◯ = very low.

## Data Availability

The data supporting the findings of this study are available within the article. All data were obtained from previously published studies cited in the reference list.
